# Delirium in Extracorporeal Membrane Oxygenation (ECMO) Patients: A Systematic Review and Meta-Analysis of Prevalence, Risk Factors, and Outcomes

**DOI:** 10.3390/jcm14248862

**Published:** 2025-12-15

**Authors:** Guangmin Yang, Johannes Greven, Sebastian Kalverkamp, Jan W. Spillner

**Affiliations:** Department of Thoracic Surgery, University Clinic RWTH Aachen, Pauwelsstraße 30, 52074 Aachen, Germany; gyang@ukaachen.de (G.Y.); jgreven@ukaachen.de (J.G.)

**Keywords:** ECMO, delirium, sedation, VA-ECMO, neurocognitive outcome, assessment tool, risk factors, heterogeneity

## Abstract

**Background**: Delirium is a common complication in patients receiving extracorporeal membrane oxygenation (ECMO), but its prevalence and determinants remain uncertain due to variable clinical practices. We conducted a systematic review and meta-analysis to estimate the pooled prevalence of delirium in ECMO-supported patients, evaluate the influence of diagnostic tools and ECMO modality, and synthesize evidence on associated risk factors and outcomes. **Methods**: We systematically searched PubMed, Embase, and Web of Science up to May 2025 for studies reporting delirium prevalence in adult ECMO patients. Subgroup analyses were stratified by ECMO modality and delirium assessment tool. Meta-regression was performed to explore potential moderators. Publication bias was assessed visually using funnel plots and statistically using Egger’s and Begg’s tests via the metabias command. **Results**: Thirteen studies involving 8679 adult ECMO patients were included. The pooled prevalence of delirium was 40.54% (95% CI: 23.01–58.06%), with substantial heterogeneity (I^2^ = 98%; τ^2^ = 0.29; *p* < 0.01). Subgroup prevalence was 56.83% (95% CI: 41.52–72.14%) for VA-ECMO, 32.84% (95% CI: 3.39–62.29%) for VV-ECMO, and 37.24% (95% CI: 13.71–60.77%) for mixed cohorts; differences were not statistically significant. Delirium prevalence varied by assessment tools, ranging from 57% with NuDESC to 7% with ICD-10 coding. Meta-regression showed a negative but non-significant association between sample size and delirium prevalence (β = −0.000049; *p* = 0.088). Sensitivity analyses confirmed the robustness of pooled estimates. **Conclusions**: Delirium affects a substantial proportion of ECMO-supported patients and is linked to considerable clinical and neurocognitive morbidity. The marked heterogeneity in prevalence reflects differences in diagnostic tools and clinical management practices, including sedation strategies. These findings underscore the urgent need for standardized, ECMO-specific delirium assessment protocols and proactive prevention strategies. Well-designed prospective studies with uniform methodologies and long-term follow-up are essential to clarify the trajectory and impact of delirium in this high-risk population.

## 1. Introduction

Extracorporeal membrane oxygenation (ECMO) is an advanced temporary cardiopulmonary support that was introduced in the late 1960s as a life-saving intervention for critically ill patients [1,2]. Over the past decades, ECMO has evolved into an indispensable rescue therapy for patients with severe respiratory and/or circulatory failure. Two principal ECMO configurations are commonly employed: veno-venous (VV) for isolated respiratory failure, and veno-arterial (VA) for combined cardiac and pulmonary support [3]. Despite its life-saving potential, ECMO carries substantial risks, including hemorrhage, thromboembolic events, neurologic injury, infection, and limb ischemia [4].

In recent years, increasing attention has been directed toward neuropsychiatric complications, particularly delirium—an acute disturbance in attention, awareness, and cognition [5]. Delirium is prevalent in critically ill patients and is associated with increased mortality, prolonged mechanical ventilation, extended ICU stay, and long-term cognitive impairment [6,7]. Its clinical presentation can be hypoactive, hyperactive, or mixed, and detection is particularly challenging in patients receiving deep sedation or neuromuscular blockade [8,9].

Although delirium has been extensively studied in general intensive care units (ICUs), its prevalence, risk factors and outcome among ECMO-supported patients remain poorly defined. This uncertainty stems in part from heterogeneous diagnostic approaches, inconsistent clinical practices, and the complex clinical profiles of ECMO recipients. These patients often require deep sedation, present with higher illness severity scores, and are exposed to multiple delirium risk factors, including prolonged mechanical ventilation, metabolic disturbances, and immobility [10,11].

A better understanding of the burden, determinants, and consequences of delirium in ECMO-supported patients is crucial for the developing tailored prevention strategies and optimizing neurocognitive monitoring in this vulnerable population. Therefore, this systematic review and meta-analysis aims to determine the overall prevalence of delirium among adult patients in the ICU and to synthesize available evidence on the influence of diagnostic tools, ECMO modality (VA vs. VV), and other clinical factors on delirium occurrence and associated outcomes.

## 2. Materials and Methods

This systematic review and meta-analysis followed the PRISMA 2020 (Preferred Reporting Items for Systematic reviews and Meta-Analyses) guideline and the MOOSE (Meta-analysis of Observational Studies in Epidemiology) statement (Supplementary File S1) [12,13]. A study protocol was not prospectively registered. The review initially began as a retrospective evidence overview, and no formal protocol existed at the time of design. After transitioning to a full systematic review, we followed PRISMA 2020 standards and documented all deviations. Planned risk-factor meta-analyses were modified post hoc due to heterogeneous data reporting. A comprehensive literature search was performed across PubMed, Embase, and Web of Science from database inception through 18 May 2025, without date restrictions. The specific search strategy for the PubMed database is provided as an example (Table S1). The search was limited to human studies published in English. Additional sources included backward citation tracking, ClinicalTrials.gov, and relevant conference proceedings to capture gray literature and unpublished studies.

### 2.1. Inclusion and Exclusion Criteria

Eligible studies met the following criteria: (1) reported original data on delirium prevalence, risk factors, or outcomes among adult ECMO patients; (2) involved veno-arterial (VA), veno-venous (VV), or mixed ECMO modalities; and (3) employed structured clinical or coded diagnostic tools to assess delirium. We included observational studies (retrospective or prospective cohorts, cross-sectional studies) and case series with ≥5 patients, in accordance with Joanna Briggs Institute (JBI) guidance for prevalence reporting [14]. Exclusion criteria included studies with pediatric-only populations, lack of delirium-specific data, use of proxy or surrogate measures without confirmation, non-English language publications, reviews, editorials, and conference abstracts.

### 2.2. Study Selection and Data Extraction

Two reviewers independently screened titles, abstracts, and full texts. Data were extracted using a standardized form, which captured study design, geographic region, sample size, ECMO modality, delirium assessment tools, prevalence, risk factors, and outcomes. Disagreements were resolved by consensus or through adjudication by a third reviewer. Study quality was assessed using the Newcastle–Ottawa Scale (NOS) for cohort and cross-sectional studies, and the JBI checklist for case series [15]. Quality grades (high/moderate/low) were derived from the NOS as follows: high-quality (≥7 stars), moderate-quality (5–6 stars), low-quality (≤4 stars). For being assessed with the JBI tool, studies meeting ≥ 80% of criteria were classified as high-quality, 60–79% as moderate, and <60% as low. Delirium was assessed using validated instruments, including the Confusion Assessment Method for the Intensive Care Unit (CAM-ICU), the Intensive Care Delirium Screening Checklist (ICDSC), the Nursing Delirium Screening Scale (NuDESC), or diagnostic codes based on the International Classification of Diseases, 10th Revision (ICD-10).

### 2.3. Data Synthesis and Statistical Analysis

Meta-analysis was performed using a random-effects model with the Freeman–Tukey double arcsine transformation via the ‘metaprop’ command in Stata 18.0 (StataCorp, College Station, TX, USA), applying exact binomial confidence intervals to stabilize variance. The primary outcome was the pooled prevalence of delirium. Heterogeneity was quantified using the I^2^ statistic, with values > 50% indicating substantial heterogeneity.

We initially planned to perform meta-analyses on delirium prevalence, risk factors, and outcomes. However, due to inconsistent and heterogeneous reporting of risk factors and outcomes across studies, we were unable to pool these data quantitatively. Therefore, risk factors and outcomes were synthesized narratively.

Subgroup analyses were performed for ECMO configuration (VA vs. VV vs. mixed) and delirium assessment tools. Meta-regression was conducted to explore heterogeneity, with sample size included as a continuous covariate. Additional moderators such as year of publication, geographic region, and assessment tool type were not included in the meta-regression because the total number of studies for each moderator was insufficient (k < 10), which is below the recommended threshold for reliable modeling. Given the small number of studies per subgroup and extreme heterogeneity, multivariable meta-regression would have been underpowered; therefore, these variables were narratively assessed with justification of methodological constraints. Sedation strategy, although clinically relevant, was also excluded from quantitative analysis due to inconsistent definitions and poor reporting.

Sensitivity analyses included leave-one-out testing and restriction to high-quality studies. Potential publication bias was assessed both visually using funnel plots and quantitatively using the ‘metabias’ command in Stata, which incorporates statistical tests such as Egger’s test or Begg’s test. No continuity correction was applied for zero-event studies, as the Freeman–Tukey method allows inclusion of 0% prevalence estimates. We also considered methodological biases arising from delirium assessment tools and study design; prior studies suggest that reliance on administrative data may underestimate prevalence, while some bedside tools may overestimate it by capturing non-specific symptoms [16,17,18].

## 3. Results

### 3.1. Study Selection and Characteristics

**Figure 1:**
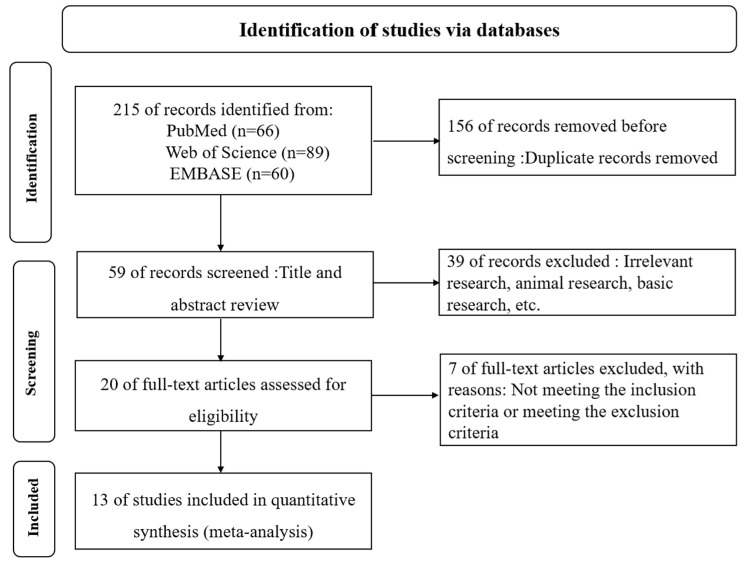
PRISMA Flow Diagram of Study Selection.

A total of 215 records were identified through systematic searches across PubMed (n = 66), Embase (n = 60), and Web of Science (n = 89). After removing 156 duplicates, 59 unique records underwent title and abstract screening. Of these, 20 full-text articles were reviewed for eligibility, and 13 studies were ultimately included in the meta-analysis (Figure 1). The included studies encompassed 8679 adult ECMO patients, with sample sizes ranging from 7 to 8153.

The study designs included 9 retrospective cohorts, 2 prospective cohorts, and 2 observational studies. Among the 13 included studies, one was rated as high quality, eight as moderate quality, and four as low quality. The detailed results of the quality assessment can be found in Table S2, and the characteristics of the included studies are summarized in Table 1. Geographically, studies were conducted in North America, Europe, Asia, and Australia. ECMO modalities varied, with three studies focused on VA-ECMO, four on VV-ECMO, and six incorporating mixed or unspecified modalities.

### 3.2. Prevalence of Delirium

The reported prevalence of delirium among ECMO patients ranged from 0% (Sklienka et al. [30]) to 84.3% (Krupa et al. [31]). The pooled prevalence, based on a random-effects meta-analysis using the Freeman–Tukey double arcsine transformation, was 40.54% (95% CI: 23.01–58.06%). Substantial heterogeneity was observed across studies (I^2^ = 98%, *p* < 0.001).

**Figure 2:**
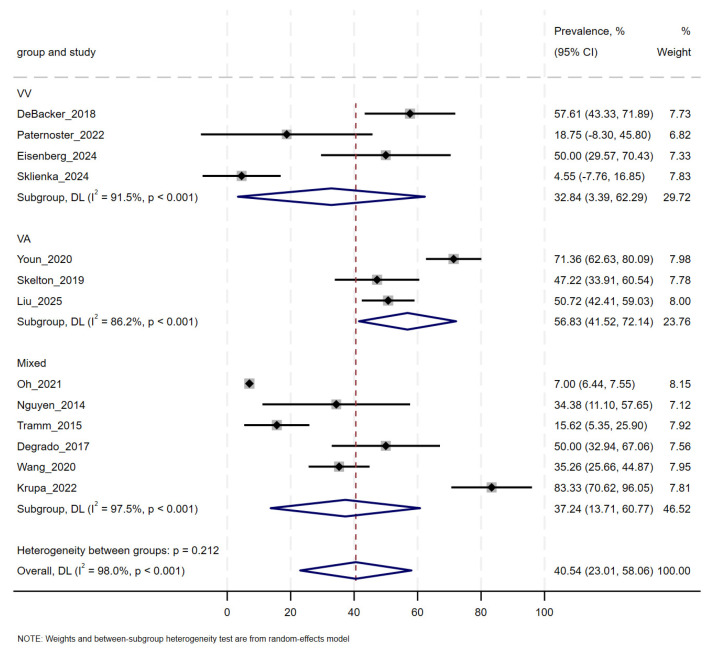
Forest Plot of Pooled Delirium Prevalence by ECMO Modality [19,20,21,22,23,24,25,26,27,28,29,30,31]. Subgroup estimates derived from Freeman–Tukey transformed random-effects model; between-group heterogeneity *p* = 0.235. Horizontal lines represent 95% confidence intervals. Summary estimates are indicated by diamonds.

In subgroup analyses by ECMO modality, the pooled prevalence was 56.83% (95% CI: 41.52–72.14%) in VA-ECMO patients, 32.84% (95% CI: 3.39–62.29%) in VV-ECMO patients, and 37.24% (95% CI: 13.71–60.77%) in mixed ECMO populations. The differences between subgroups were not statistically significant (*p* = 0.212). High heterogeneity was present within all subgroups (Figure 2).

### 3.3. Influence of Assessment Tools

The reported prevalence of delirium varied substantially depending on the assessment tool used (Table 2). Among the 13 included studies, nine used the Confusion Assessment Method for the ICU (CAM-ICU), two employed the Intensive Care Delirium Screening Checklist (ICDSC), three applied the Nursing Delirium Screening Scale (NuDESC), and one relied on administrative coding using the ICD-10. The pooled prevalence of delirium was 57% (95% CI: 44–68%) in NuDESC-based studies, 51% (95% CI: 47–55%) in CAM-ICU studies, and 41% (95% CI: 32–50%) in ICDSC studies. The single study that utilized ICD-10 discharge codes reported a considerably lower prevalence of 7%. One NuDESC-based study (Sklienka et al. [30]) reported 0% prevalence and was excluded from the pooled estimate due to its small sample size (n = 10), although its findings were included in the descriptive summary.

### 3.4. Risk Factors for Delirium

Across the included studies, several clinical outcomes were reported in association with delirium, although definitions and reporting frameworks varied substantially. When data were available, delirium was consistently associated with prolonged ICU length of stay, longer duration of mechanical ventilation, increased antipsychotic use, and a higher likelihood of discharge to rehabilitation facilities rather than home. However, only a minority of studies stratified outcomes by delirium status, and the specific operational definitions of each outcome differed considerably. Owing to these inconsistencies and the limited availability of comparable numerical data, these outcomes could not be pooled in a formal meta-analysis. Instead, they were synthesized qualitatively to reflect the direction and consistency of observed associations.

Risk factors for delirium were similarly heterogeneous in reporting. While advanced age, deeper or prolonged sedation, VA-ECMO configuration, longer pre-ECMO mechanical ventilation, and longer hospitalization were commonly associated with delirium, the definitions, measurement methods, and effect sizes varied across studies. As a result, these risk factors were narratively synthesized rather than quantitatively aggregated.

Delirium in ECMO patients was influenced by a combination of modifiable and non-modifiable factors, as reported across the included studies. Among modifiable factors, sedation practices were the most frequently described contributors. Deep or prolonged sedation, particularly involving benzodiazepines, was associated with higher delirium prevalence in multiple studies [20,23,24,28] (Table 3). In contrast, studies that implemented lighter sedation regimens or daily awakening protocols reported relatively lower rates [20,21], though heterogeneity in sedation strategies and illness severity limited direct comparison.

Early mobilization and awake ECMO protocols were described as potential protective strategies in selected cohorts. Paternoster et al. [21] reported lower delirium occurrence in non-intubated, awake VV-ECMO patients, while Eisenberg et al. [29] noted improved functional outcomes in patients with structured rehabilitation. However, these findings were derived from small or observational studies and were mainly applied in patients with less severe disease.

Among non-modifiable factors, advanced age, baseline cognitive impairment, and ECMO modality were commonly identified in the included literature [22,23,24]. VA-ECMO was associated with higher delirium prevalence compared to VV-ECMO in several studies [22,23,31]. Youn et al. reported a delirium prevalence of 71.5% in VA-ECMO patients [22], and Krupa et al. observed a 84.3% delirium incidence in a mixed cohort with predominant VA-ECMO support [31].

### 3.5. Clinical Outcomes and Evidence Gaps

Short-term outcomes associated with delirium were reported in several studies (Table 4). Youn et al. [22] found that patients with delirium had a longer median ICU stay (18.5 vs. 12.0 days, *p* < 0.05) and prolonged mechanical ventilation duration compared to those without delirium. Wang et al. [28] reported increased haloperidol use (67% vs. 20%) and higher Richmond Agitation-Sedation Scale (RASS) scores among patients diagnosed with hyperactive delirium. Eisenberg et al. [29] observed that delirious patients had slower post-ECMO functional recovery and were more frequently discharged to rehabilitation facilities (82% vs. 45%). They found that patients who experienced delirium during ECMO had continued rehabilitation needs after discharge. No included studies systematically assessed long-term cognitive function, neuropsychological outcomes, or caregiver burden. Evidence on long-term outcomes of delirium in ECMO patients remains limited.

### 3.6. Meta-Regression and Sensitivity Analyses

Substantial heterogeneity was present across studies (τ^2^ = 0.29; Q-test *p* < 0.01). The 95% prediction interval ranged from 10.6% to 76.3%, indicating that true delirium prevalence may vary widely across clinical settings.

Meta-regression was performed using sample size as a continuous covariate. The analysis demonstrated a negative but non-significant association between sample size and reported delirium prevalence (β = −0.000049; 95% CI: −0.000107 to 0.000009; *p* = 0.088), suggesting that studies with larger sample sizes tended to report lower prevalence estimates, although this association did not reach statistical significance. Sensitivity analyses supported the robustness of the main pooled estimate.

**Figure 3:**
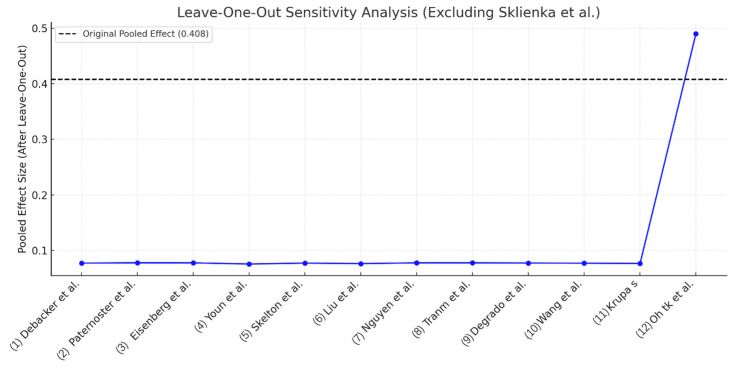
Leave-one-out sensitivity analysis for delirium prevalence in ECMO patients [19,20,21,22,23,24,25,26,27,28,29,30,31]. The plot shows the impact of sequentially omitting each study on the pooled prevalence estimate (back-transformed from Freeman–Tukey transformation). Sklienka et al. (2024) [30] was excluded from this analysis because it reported zero delirium event in a very small and highly selected cohort awake VV-ECMO cohort (n = 10); inclusion of such a study may introduce numerical instability without meaningfully informing influence diagnostics. Overall, the pooled prevalence estimate remained stable across study exclusions, with the greatest change observed following omission of Oh et al. (2022) [19], a large administrative dataset relying on ICD-10-code delirium diagnoses.

Leave-one-out analyses (Figure 3) showed that exclusion of any single study yielded back-transformed pooled prevalence estimates ranging from 38.4% to 44.2%. Removal of the largest study, Oh et al., 2022 [19]—a large administrative dataset of 8153 patients with a reported delirium prevalence of 6.9%—increased the pooled prevalence from 40.8% to 49.1%, demonstrating its substantial down-weighting influence on the overall estimate because of reliance on ICD-coded delirium definitions.

Publication bias was evaluated using both visual and quantitative approaches. Visual inspection of the funnel plot of Freeman–Tukey–transformed prevalence estimates suggested some asymmetry; however, interpretation was limited by the small number of studies. To complement visual assessment, Egger’s and Begg’s statistical tests were performed using the metabias command. Egger’s regression test indicated significant asymmetry (bias = 5.91, *p* = 0.001), suggesting the presence of publication bias, whereas Begg’s rank correlation test did not detect significant asymmetry (*p* = 0.329). Given that Egger’s test generally has greater statistical power than Begg’s test in small-sample meta-analyses, these results should be interpreted cautiously, and evidence of publication bias cannot be ruled out.

## 4. Discussion

This meta-analysis of 13 studies involving 8679 ECMO-supported adult patients demonstrates that delirium is a frequent and clinically important complication, with a pooled prevalence of 40.54% (95% CI: 23.01–58.06%) and a wide range across studies (0–84%). The observed prevalence appears higher than typically reported in general ICU populations (20–30%) [6,7], suggesting that ECMO recipients have heightened neuropsychiatric vulnerability. Subgroup analysis showed numerically higher prevalence in VA-ECMO patients (56.83%) compared to VV-ECMO (32.84%) or mixed cohorts (37.24%); however, these differences were not statistically significant and were accompanied by substantial heterogeneity (I^2^ = 98%, *p* < 0.001). The non-significant subgroup difference should not be interpreted as evidence of equivalence; however, it likely reflects limited statistical power due to the small number of studies available for each ECMO modality, coupled with extreme heterogeneity. Regional variations in ECMO utilization patterns, sedation practices, and staffing ratios may also contribute to the observed heterogeneity, underscoring the need for context-specific delirium prevention strategies.

Several factors may contribute to this elevated risk. Advanced age and greater illness severity were consistently associated with delirium in the included studies, reflecting reduced neuroplasticity, increased susceptibility to systemic inflammation, and pre-existing cognitive impairment [32,33]. Patients supported with VA-ECMO often present with profound hemodynamic instability, systemic hypoperfusion, and multi-organ dysfunction—conditions known to precipitate delirium [10,34]. Moreover, the non-physiological circulatory pattern of VA-ECMO, characterized by reduced or absent pulsatility and the potential for differential hypoxia (Harlequin syndrome), can directly impair cerebral autoregulation and produce unstable, pressure-passive cerebral perfusion. These alterations may further compromise cortical oxygenation and metabolism, consistent with findings from neuroimaging and preclinical studies showing impaired cerebral autoregulation and elevated neurological risk in VA-ECMO patients [34,35,36,37,38,39].

These hemodynamic disturbances occur alongside ECMO-related pharmacologic perturbations. The ECMO circuit is known to sequester lipophilic sedatives such as fentanyl and midazolam, increasing their volume of distribution and altering clearance, which complicates sedation management and may lead to unpredictable sedation depth or the development of delirium [34]. Together, these acute abnormalities in cerebral perfusion and drug pharmacokinetics may serve as early triggers for the high burden of long-term neuropsychiatric symptoms, neurocognitive deficits, and functional impairment documented in ECMO survivors.

Furthermore, delirium may interact bidirectionally with other ECMO-related complications—including ischemic stroke, intracranial hemorrhage, and prolonged mechanical ventilation—thereby amplifying their effects on neurological and functional outcomes [34]. Prolonged ICU or hospital stay also represents an independent risk factor, as extended critical illness increases exposure to deep sedation, immobility, circadian disruption, and inflammatory stress. Evidence from both ECMO and non-ECMO ICU cohorts demonstrates a strong association between length of hospitalization and delirium incidence, suggesting that cumulative exposure to the ICU environment heightens neurocognitive vulnerability [19,40]. These processes—from acute cerebrovascular instability and altered sedative pharmacokinetics, to delirium and subsequent long-term cognitive decline—reflect the “ICU Brain Failure Continuum” as it manifests in ECMO-supported patients [41].

Although sedation strategy was not assessed quantitatively in our meta-regression due to inconsistent reporting, narrative synthesis indicated that deep or prolonged sedation—particularly with benzodiazepines—was frequently associated with higher delirium prevalence [20,23,24,28]. ECMO circuits can sequester sedatives, alter pharmacokinetics, and promote delayed drug accumulation [42,43], potentially prolonging sedation and impairing cognition. These pharmacokinetic alterations have been demonstrated in studies showing marked reductions in circulating sedative concentrations during ECMO [34], underscoring the importance of individualized dosing and close monitoring.

The variation in reported delirium prevalence was also strongly influenced by the choice of assessment tool. In the included studies, NuDESC yielded the highest pooled prevalence (57%), followed by CAM-ICU (51%) and ICDSC (41%), whereas ICD-10 coding produced substantially lower rates (7%). In a sensitivity analysis excluding the large administrative study that used ICD-10 coding, the pooled prevalence increased to approximately 49%, suggesting that the clinical prevalence detected by bedside assessment is considerably higher. These differences parallel findings in general ICU populations and highlight the challenges of delirium detection in ECMO patients. Deep sedation, neuromuscular blockade, and limited patient responsiveness hinder the application of standard screening tools, particularly for hypoactive delirium, which is common but often underrecognized. CAM-ICU is validated and widely used but less sensitive in deeply sedated patients (RASS ≤ −3) [16,17], whereas NuDESC may detect hypoactive features but risks overestimating prevalence due to nonspecific symptom inclusion [29,31]. Administrative coding underestimates delirium prevalence, especially for hypoactive and subsyndromal forms [17,19]. Improving detection will require structured, protocolized monitoring tailored to sedation level, potentially integrating behavioral assessment tools with neurophysiological modalities such as EEG to enhance diagnostic accuracy. Incorporating multimodal neuromonitoring—such as continuous EEG, near-infrared spectroscopy, and transcranial Doppler—may enable earlier detection of brain dysfunction and facilitate individualized sedation and perfusion strategies aimed at reducing delirium risk [44].

From a clinical perspective, preventive and management strategies for delirium in ECMO patients should adapt principles from established ICU protocols while evolving toward ECMO-specific prevention bundles that integrate sedation minimization, early mobilization, structured sleep promotion, and continuous neurologic monitoring, as recently emphasized in updated critical care guidelines. Light sedation regimens, daily awakening trials, early mobilization, sleep hygiene, and cognitive re-orientation have been associated with reduced delirium in general critical care [17,18] and are applicable, with modification, to ECMO populations. These approaches can be aligned with established ICU care frameworks—such as principles derived from the ABCDEF bundle—when adapted appropriately to the ECMO setting [45], and future work should focus on developing ECMO-tailored versions of this bundle [18]. “Awake ECMO” protocols represent a promising but underutilized approach, enabling earlier mobilization and more reliable neurologic assessment [21]; however, current evidence is limited to small observational series and requires confirmation in multicenter, prospective studies employing standardized assessment intervals and multimodal neuromonitoring. Sedation protocols should consider ECMO-related pharmacokinetic changes, implement drug-level monitoring when feasible, and adjust dosing dynamically to avoid excessive accumulation and delayed emergence. In this context, sedation strategies favoring dexmedetomidine or emerging agents such as remimazolam—whose pharmacokinetics may offer advantages in minimizing delirium—warrant further investigation [42]. Given the resource-intensive nature of ECMO care, delirium may further prolong ICU stay, increase rehabilitation needs, and elevate post-discharge care costs, highlighting the importance of cost-effective prevention and management strategies.

This review has several limitations. The absence of prospective protocol registration may introduce analytic bias. Furthermore, the pooled estimate was heavily influenced by one large administrative database study, which comprised 93.9% of the total sample but likely underestimated the true prevalence due to reliance on diagnostic coding. Sedation strategy, a key modifiable factor, could not be quantitatively analyzed due to inconsistent and incomplete reporting. The predominance of retrospective, single-center studies limit causal inference, and small sample sizes and selection bias may have influenced prevalence estimates. The administrative study by Oh et al. [19] contributed over 90% of the pooled sample size. Although leave-one-out sensitivity analysis indicated that the overall findings were robust, the weighting effect of this large dataset may still bias the pooled prevalence estimate download. Diagnostic tools, assessment timing, and reporting methods varied widely, contributing to substantial heterogeneity. Database-derived ICD-coded delirium may substantially underestimate prevalence due to coding insensitivity, lack of standardized screening, and potential misclassification. Delirium during ECMO may predispose survivors to persistent cognitive impairment, mood disorders, and reduced health-related quality of life, reinforcing the need for longitudinal follow-up and targeted rehabilitation programs. Furthermore, long-term neurocognitive outcomes were rarely assessed, restricting understanding of the lasting impact of delirium in ECMO survivors.

## 5. Conclusions

Delirium is a common but variably reported complication among ECMO patients, with an overall prevalence exceeding 40%. When assessed prospectively at the bedside, its prevalence may be considerably higher, approaching 50% or more. Assessment tool choice and sample size significantly influence reported rates, while ECMO modality may interact with patient characteristics and management strategies to modulate risk. Implementation of standardized bedside tools, coupled with preventive interventions and long-term follow-up, is essential to mitigate the neurocognitive burden of ECMO-related delirium. Future subgroup analyses with larger samples and standardized reporting may help clarify interactions between ECMO modality, sedation strategies, and delirium risk. Future prospective studies should adopt harmonized diagnostic frameworks, stratify by delirium subtype, and include structured reporting of outcome measures to better quantify the impact of delirium on clinical trajectories.

## Figures and Tables

**Table 1:** Characteristics of Included Studies.

First Author (Year)	Country	Design. Sample Size	Delirium Cases/Prevalence (%)	Mean Age	ECMO Indication	ECMO Duration (Days)	Delirium Assessment Tool	ECMO Type	Quality Grade	Conclusion
Oh et al. (2022) [19]	South Korea	Retrospective, 8153	570 (6.9)	52	Mixed	6.7	ICD-10	Mixed	High	Large administrative cohort showed low reported delirium prevalence (6.9%) based on ICD-10 coding, suggesting potential underdetection.
Debacker et al. (2018) [20]	Canada	Retrospective, 45	26 (57.7)	47 (IQR 35–56)	Respiratory failure	NR	CAM-ICU and ICDSC	VV	Moderate	Over half of VV-ECMO patients developed delirium; deep sedation was common after ECMO initiation.
Paternoster et al. (2022) [21]	Italy	Observational, 7	1 (14.2)	51.7 ± 12.5	COVID-19 ARDS	Median15 (IQR 2–71)	CAM-ICU	VV	Low	Awake, non-intubated VV-ECMO was feasible in selected COVID-19 patients; delirium occurred in one case and contributed to ECMO termination.
Youn et al. (2020) [22]	South Korea	Retrospective, 102	73 (71.5)	57	Cardiac surgery	Mediana 10.0 (IQR 4.3–17.3)	CAM-ICU	VA	Moderate	High delirium prevalence (71.5%) observed in post-cardiac surgery VA-ECMO patients, highlighting a vulnerable subgroup.
Skelton et al. (2020) [23]	UK	Retrospective, 53	25 (47.2)	63	Cardiogenic shock	6.2	CAM-ICU	VA	Moderate	Nearly half of VA-ECMO patients developed delirium; all cases occurred during or after periods of deep sedation.
Liu et al. (2025) [24]	China	Retrospective, 138	70 (50.7)	55	Respiratory failure	7.2	CAM-ICU	VA	Moderate	Moderate delirium rate (50.7%) reported in VA-ECMO patients with respiratory failure; no association analysis provided.
Nguyen et al. (2014) [25]	Belgium	Prospective, 15	5 (33.3)	58	Cardiogenic shock, aspiration pneumonia	7.7	CAM-ICU	Mixed	Moderate	One-third of patients experienced delirium; authors emphasized the need for systematic monitoring in ECMO settings.
Tramm et al. (2015) [26]	Australia	Retrospective, 47	7 (14.8)	60	Respiratory failure, cardiac arrest	NR	CAM-ICU	Mixed	Low	Delirium occurred in 14.8% of patients receiving prolonged ECMO; prevalence may be underestimated due to retrospective design.
DeGrado et al. (2017) [27]	USA	Prospective, 32	16 (50.0)	62.5	Cardiac surgery	6.5	CAM-ICU	Mixed	Moderate	50% of patients developed delirium; authors highlighted the impact of sedatives and neuromuscular blockers.
Wang et al. (2020) [28]	China	Retrospective, 94	33 (35.1)	59 (IQR 51–67)	Mixed	5.0	ICDSC and CAM-ICU	Mixed	Moderate	Delirium was present in over one-third of patients; benzodiazepine use and hyperactive symptoms were associated with increased risk.
Eisenberg et al. (2024) [29]	USA	Retrospective, 22	11 (50.0)	NR	Severe respiratory failure	Mean 23 (delirium) vs. 15 (non-delirium)	NuDESC	VV	Moderate	50% delirium prevalence post-ECMO; delirium was associated with longer ECMO runs, greater antipsychotic use, and increased rehabilitation needs.
Sklienka et al. (2024) [30]	Czech Republic	Retrospective, 10	0	55 ± 12	COVID-19	23.3 ± 7.2 *	NuDESC	VV	Low	No delirium reported in small awake VV-ECMO cohort; results limited by sample size and retrospective nature.
Krupa et al. (2021) [31]	Poland	Cross-sectional, 32	27 (84.3)	NR	Cardiac surgery	NR	NuDESC	Mixed	Low (JBI)	High delirium prevalence (84.3%) in mixed ECMO cohort dominated by VA-ECMO; NuDESC identified mostly hypoactive cases.

* Converted when possible; NR: Not reported.

**Table 2:** Delirium Prevalence by Assessment Tool Used Across Studies.

Assessment Tool	Pooled Prevalence (%)	95% CI	Total Sample Size	No. of Studies
CAM-ICU	51	47–55%	489	9
ICDSC	41	32–50%	139	2
NuDESC	57	44–68%	64	3
ICD-10	7	6–8%	8153	1

One study [20] and another [28] employed both CAM-ICU and ICDSC but were categorized under both tools accordingly for pooled analysis. Ref. [30], which used NuDESC and reported 0% prevalence, was excluded from pooled prevalence estimation due to the extreme value and limited sample size (n = 10), but included in the count of NuDESC users. CI: confidence interval; CAM-ICU: Confusion Assessment Method for the ICU; ICDSC: Intensive Care Delirium Screening Checklist; NuDESC: Nursing Delirium Screening Scale.

**Table 3:** Qualitative Summary of Sedation Practices and Association with Delirium in ECMO Patients.

Risk Factor	Direction of Effect	Evidence Base and Explanations
Deep Sedation	Increased Risk	Debacker et al. [20]: Patients were deeply sedated for the majority of the initial ECMO period, with a concurrent high delirium incidence of 58%. Liu et al. [24]: Delirium patients had more coma days, suggesting a strong link between deep sedation and delirium (or its masking).
Benzodiazepine Use	Increased Risk	Liu et al. [24]: The “number of sedatives” (primarily benzodiazepines) was an independent risk factor for delirium (aOR = 2.67). Skelton et al. [23]: A ≥50% reduction in benzodiazepines post-decannulation did not significantly reduce delirium incidence compared to a <50% reduction, but the authors noted that a 50% reduction may be insufficient to mitigate the risk. Wang et al. [28]: The agitation group had a higher proportion of midazolam use on ECMO day 2 (potentially a consequence of managing agitation, but reflects a strong association with adverse neurological states).
Sedative Polypharmacy	Increased Risk	Liu et al. [24]: Delirium patients received a significantly greater total number of analgesic-sedative drugs, and the number of sedatives was an independent predictor of delirium (aOR = 2.67).
Dexmedetomidine	Potential Protective Effect	Wang et al. [28]: The non-agitation group had a significantly higher proportion of dexmedetomidine use on ECMO day 2, indicating its potential protective role as an alternative sedative.
Ketamine	Potential Protective	Skelton et al. [23]: Patients receiving ketamine as an adjunctive sedative had a numerically lower incidence of delirium (0% vs. 14%), though it was not statistically significant, likely due to small sample size.
Early Empirical Sedation Reduction	Inconclusive Effect	Skelton et al. [23]: Empirically reducing benzodiazepines by 50% or more immediately after ECMO decannulation did not significantly reduce subsequent delirium incidence. This suggests the need for more aggressive reduction protocols or optimized pre-weaning sedation strategies.

**Table 4:** Summary of Clinical Outcomes in ECMO Patients with and Without Delirium.

Study	Compared Groups	Outcomes Impacted	Findings
Youn et al. [22]	Delirium vs. non-delirium	ICU stay, mechanical ventilation	Longer ICU stay and MV duration in delirium group
Wang et al. [28]	Agitation (delirium) vs. control	Haloperidol use, MV duration	Higher haloperidol use, longer MV
Eisenberg et al. [29]	Delirium vs. non-delirium	Rehab need, recovery time	More rehab transfer, slower recovery

ICU, intensive care unit; MV, mechanical ventilation.

## Data Availability

The data supporting the findings of this study are available within the article and its Supplementary Materials. The Stata analysis code is available from the corresponding author upon reasonable request.

## Supplementary Materials

The following supporting information can be downloaded at https://www.mdpi.com/article/10.3390/jcm14248862/s1, Table S1: PubMed/Embase/Web of Science Search History; Table S2: Methodological Quality Assessment of Included Studies Using the Joanna Briggs Institute (JBI) Critical Appraisal Tools; File S1: PRISMA 2020 checklist.
