# Supplementary material for: Delirium in Extracorporeal Membrane Oxygenation (ECMO) Patients: A Systematic Review and Meta-Analysis of Prevalence, Risk Factors, and Outcomes
Source: J Clin Med. 2025 Dec 15;14(24):8862. doi: 10.3390/jcm14248862 (PMC12734077; doi:10.3390/jcm14248862)
Supplement: Supplementary file 1 [file jcm-14-08862-s001.zip › jcm-3967937-tables.pdf]

Journal of Clinical Medicine

MDPI

Table S1. PubMed/Embase/Web of Science Search History.

Search number	Query	Results
**Pubmed**		
#1	"Delirium"[Mesh]	14,761
#2	(((((((Delirium of Mixed Origin) OR (Mixed Origin Delirium)) OR (Mixed Origin Deliriums)) OR (Subacute Delirium)) OR (Deliriums, Subacute)) OR (Delirium, Subacute)) OR (Subacute Deliriums)) OR (Acute Confusional State)) OR (ICU Delirium)	33,791
#3	(#1) OR (#2)	33,791
#4	"Extracorporeal Membrane Oxygenation"[Mesh]	17,967
#5	(((((((((((((((((((Extracorporeal Membrane Oxygenations) OR (Membrane Oxygenation, Extracorporeal)) OR (ECMO Extracorporeal Membrane Oxygenation)) OR (Extracorporeal Life Support)) OR (Extracorporeal Life Supports)) OR (Life Support, Extracorporeal)) OR (ECMO Treatment)) OR (ECMO Treatments)) OR (Treatment, ECMO)) OR (ECLS Treatment)) OR (ECLS Treatments)) OR (Treatment, ECLS)) OR (Oxygenation, Extracorporeal Membrane)) OR (Venoarterial ECMO)) OR (ECMO, Venoarterial)) OR (Venoarterial ECMOs)) OR (Venoarterial Extracorporeal Membrane Oxygenation)) OR (Venovenous ECMO)) OR (ECMO, Venovenous)) OR (Venovenous ECMOs)) OR (Venovenous Extracorporeal Membrane Oxygenation)) OR (ECMO)	31,516
#6	(#4) OR (#5)	31,516
#7	(#3) AND (#6)	102
#8	((((case reports [Publication Type]) OR (letter [Publication Type])) OR (editorial)) OR (comment)) OR (interview)	5,037,258
#9	(#7) NOT (#8)	66
**Embase**		
#3	#1 AND #2	
#2	'extracorporeal membrane oxygenation':ab,ti OR 'extracorporeal membrane oxygenations':ab,ti OR 'membrane oxygenation, extracorporeal':ab,ti OR 'ecmo extracorporeal membrane oxygenation':ab,ti OR 'extracorporeal life support':ab,ti OR 'extracorporeal life supports':ab,ti OR 'life support, extracorporeal':ab,ti OR 'ecmo treatment':ab,ti OR 'ecmo treatments':ab,ti OR 'treatment, ecmo':ab,ti OR 'ecls treatment':ab,ti OR 'treatment, ecls':ab,ti OR 'oxygenation, extracorporeal membrane':ab,ti OR 'venoarterial ecmo':ab,ti OR 'ecmo, venoarterial':ab,ti OR 'venoarterial ecmos':ab,ti OR 'venoarterial extracorporeal membrane oxygenation':ab,ti OR 'venovenous ecmo':ab,ti OR 'ecmo, venovenous':ab,ti OR 'venovenous ecmos':ab,ti OR 'venovenous extracorporeal membrane oxygenation':ab,ti OR 'ecmo':ab,ti	
#1	'delirium':ab,ti OR 'delirium of mixed origin':ab,ti OR 'mixed origin delirium':ab,ti OR 'mixed origin deliriums':ab,ti OR 'subacute delirium':ab,ti OR 'deliriums, subacute':ab,ti OR 'delirium, subacute':ab,ti OR 'subacute deliriums':ab,ti OR 'acute confusional state':ab,ti OR 'icu delirium':ab,ti	
**Web of Science** Strings	((TS=(Delirium OR Delirium of Mixed Origin OR Mixed Origin Delirium OR Mixed Origin delirium OR Subacute Delirium OR delirium, Subacute OR Delirium, Subacute OR Subacute delirium OR Acute Confusional State OR ICU Delirium)) AND	

	TS=(Extracorporeal Membrane Oxygenation OR Extracorporeal Membrane oxygenation OR Membrane Oxygenation, Extracorporeal OR ECMO Extracorporeal Membrane Oxygenation OR Extracorporeal Life Support OR Extracorporeal Life Supports OR Life Support, Extracorporeal OR ECMO Treatment OR ECMO Treatments OR Treatment, ECMO OR ECLS Treatment OR ECLS Treatments OR Treatment, ECLS OR Oxygenation, Extracorporeal Membrane OR vivoarterial ECMO OR ECMO, vivoarterial OR vivoarterial echos OR vivoarterial Extracorporeal Membrane Oxygenation OR Venovenous ECMO OR ECMO, Venovenous OR Venovenous echos OR Venovenous Extracorporeal Membrane Oxygenation OR ECMO)) NOT TS=(case reports OR letter OR editorial OR comment OR interview)

Table S2. Methodological Quality Assessment of Included Studies Using the Joanna Briggs Institute (JBI) Critical Appraisal Tools.

**Study**	**Criteria**							
	a	b	c	d	e	f	g	h
Oh et al19. (2022)	Yes	Yes	Yes	Yes	Yes	Yes	Yes	Yes
Debacker et al20. (2018)	Yes	Yes	Yes	Yes	Yes	No	Unclear	Yes
Paternoster et al21. (2022)	Unclear	No	Unclear	Yes	No	Not Applicable	Yes	No
Youn et al22. (2020)	Yes	Yes	Yes	Yes	Unclear	Yes	Yes	No
Skelton et al23. (2020)	Yes	Yes	Yes	Yes	No	Not Applicable	Yes	Yes
Liu et al24. (2025)	Yes	Unclear	Yes	Yes	Yes	No	Yes	Yes
Nguyen et al25. (2014)	Yes	No	Yes	Yes	Yes	Unclear	Yes	Yes
Tramm et al26. (2015)	Yes	Unclear	Yes	Unclear	No	No	Unclear	Unclear
Degrado et al27. (2017)	Yes	Yes	Unclear	Yes	Yes	Yes	Yes	Yes
Wang et al28. (2020)	Yes	Yes	No	Unclear	Yes	Yes	Yes	Yes
Eisenberg et al29. (2024)	Yes	Yes	Yes	Yes	Yes	Yes	Unclear	Yes
Sklienka et al30. (2024)	Unclear	No	Yes	Yes	No	Not Applicable	Yes	No
Krupa et al31. (2021)	Yes	Yes	No	Unclear	No	Not Applicable	Yes	No

(a) Were the criteria for inclusion in the sample clearly defined? (b) Were the study subjects and the setting described in detail? (c) Was the exposure measured in a valid and reliable way? (d) Were objective, standard criteria used for measurement of the condition? (e) Were confounding factors identified? (f) Were strategies to deal with confounding factors stated? (g) Were the outcomes measured in a valid and reliable way? (h) Was appropriate statistical analysis used?
